# A Comparative Study of Dogs and Goats with Limited Human Socialization in the Impossible Task Paradigm

**DOI:** 10.3390/ani13193027

**Published:** 2023-09-26

**Authors:** Alfredo Di Lucrezia, Anna Scandurra, Claudia Pinelli, Nadia Musco, Biagio D’Aniello, Vincenzo Mastellone, Fabio Zicarelli, Maria Elena Pero, Pietro Lombardi

**Affiliations:** 1 Department of Biology, University of Naples Federico II, 80126 Naples, Italy; alfredo.dilucrezia@unina.it (A.D.L.); anna.scandurra@unina.it (A.S.); biagio.daniello@unina.it (B.D.); 2 Department of Environmental, Biological and Pharmaceutical Sciences and Technologies, University of Campania “Luigi Vanvitelli”, 81100 Caserta, Italy; 3 Department of Veterinary Medicine and Animal Production, University of Naples Federico II, 80137 Naples, Italy; nadia.musco@unina.it (N.M.); vincenzo.mastellone@unina.it (V.M.); mp3054@cumc.columbia.edu (M.E.P.); pilombar@unina.it (P.L.); 4 Department of Health Sciences, University Magna Graecia, Viale Europa, 88100 Catanzaro, Italy; fabio.zicarelli@gmail.com; 5 Department of Pathology, Anatomy and Cell Biology, Columbia University, New York, NY 10032, USA

**Keywords:** comparative, dog, goat, impossible task, human–animal interaction, interspecific communication, under socialization

## Abstract

**Simple Summary:**

This research investigates the influence of limited human socialization on the socio-cognitive abilities and interactions with unfamiliar individuals of a selected group of domesticated dogs and goats. Both dogs and goats were raised and kept under conditions with limited human socialization, and their behavior was examined through the “impossible task” paradigm. Dogs exhibited higher interaction frequencies with human experimenters compared to goats, indicating a potential inclination for engaging with humans. However, no significant differences were observed in interaction duration and latency, underscoring the intricate nature of these interactions. This study underscores the necessity of considering the multifaceted aspects of social behavior and cognition, especially within domesticated animals characterized by diverse behavioral ecologies and domestication histories.

**Abstract:**

The study aimed to explore how limited human socialization affects the socio-cognitive abilities and interactions with unfamiliar individuals of a selected group of domesticated dogs and goats. These animals were raised and kept under conditions characterized by limited human socialization, and their behavior was assessed using the “impossible task” paradigm. The study found that dogs, with a history of cooperative interactions and human companionship, exhibited more frequent social engagement with human experimenters in the experimental setting than goats, traditionally domesticated for utilitarian purposes. However, differences in interaction duration and latency were not significant, highlighting the complexity of these interactions. The results suggest that domestication history and behavioral ecology play significant roles in shaping animals’ willingness to engage with humans. However, this study acknowledges limitations, such as the specific population studied, and calls for further research with larger and more diverse samples to generalize these findings. Understanding the interplay between domestication history, behavioral ecology, and human socialization could provide insights into the complex factors influencing animal–human interactions and cognitive behaviors, with implications for animal welfare and human–animal relationships.

## 1. Introduction

The fitness of domesticated animal species is under human control, leading to noticeable differences in their physical, genetic, and behavioral characteristics compared to their wild ancestors [1]. Behavioral changes in domesticated animals can be attributed, in part, to the alterations in their socio-cognitive abilities, making them more skilled at interacting with humans [2,3], probably as a result of selective pressure favoring tameness during domestication [4]. Interestingly, similar effects on socio-cognitive abilities have been observed in certain wild animal populations that have adapted to the anthropogenic niche [5].

Animal domestication has been driven by various purposes and influenced by the behavioral ecology of ancestral species. This suggests that the behavioral changes observed in domesticated animals, aimed at enhancing their positive interaction with humans, might have followed different routes. Furthermore, the process of experiential learning, particularly socialization in a human environment during ontogenesis, can significantly influence the socio-communicative abilities of animals and subsequently affect the way they interact with humans [6,7]. Therefore, understanding the behavior of domesticated animals towards humans requires a comprehensive consideration of various aspects, including the context of domestication, the behavioral ecology of ancestor species, and the ontogenetic factors, making it challenging to isolate the individual relative effects of these components.

Despite the extensive body of research on the cognitive abilities of domesticated animals, there remains a scarcity of direct comparative evaluations between different species within the same study. In an effort to address this, researchers have conducted a study that aimed to assess the cue-following ability of dogs and horses, investigating how these two species respond to human gestures. This study’s results indicated that dogs displayed a significantly higher level of skill in following human gestures compared to horses [8]. In a more recent study by Gerencsér et al. [9], spontaneous reactions of miniature pigs and family dogs towards humans were compared at an early age of 4 months. Both species exhibited a spontaneous emergence of similar socio-communicative behaviors. However, dogs demonstrated a higher propensity to make eye contact with humans in comparison to miniature pigs. Additionally, this study explored the responses of both species to the distal dynamic-sustained pointing gesture, revealing that only dogs responded above the chance level.

The impossible task paradigm is a valuable protocol for investigating animal–human interactions. This approach involves presenting animals with solvable tasks where they learn to complete a simple task in exchange for a reward. However, at a certain point, the reward becomes unreachable, creating an expectation violation. The presence of humans during the test allows researchers to observe the animals’ interactions and communication, providing valuable insights into their persistence in solving the task or to switch to a social strategy [10]. Nevertheless, considering that the task essentially mimics a foraging activity, the influences of the species’ foraging ecology should not be overlooked when drawing broader conclusions.

Using this experimental paradigm, researchers have identified notable differences between dogs and cats. Cats have been observed to display a higher persistence in completing tasks and exhibit less interactions with humans compared to dogs [11]. In another study, the human-directed behaviors of pigs and dogs, both approximately 7 months old, were compared [12]. The findings revealed that during the unsolvable phase of the task, dogs exhibited human-directed behaviors more frequently than pigs. On the other hand, pigs spent more time interacting with the apparatus compared to the dogs. It is essential to mention that in the aforementioned studies, piglets, puppies, dogs, and cats were all socialized in human families or were family pets [9,11,12]. These factors can affect the interaction between animals and humans, as demonstrated with dogs [13,14]. By controlling for human socialization, both studies converge in emphasizing the significance of the behavioral ecology of the ancestors of domesticated animals, as well as the impact of the domestication process in shaping animal interactions with humans.

In the context of the impossible task, it has been observed that ontogenetic factors can have a significant influence on the results. Indeed, training can affect the behavioral outcome in dogs [15,16,17], as well as the human socialization in both dogs [18,19] and goats [20,21]. In all the aforementioned comparative studies [8,9,11,12], animals underwent significant human socialization and were tested in the presence of familiar people. The perception and expectations humans hold regarding different animal species can significantly influence the dynamics of their interactions. For instance, cat owners often admire their pets for their independent and self-reliant personalities [22], which might not be the primary appeal for dog owners. Indeed, relationships with dogs can be different from those with cats [23,24]. These preconceived notions and preferences about various animal species can impact how people engage and communicate with them, subsequently influencing the behavior of the animals. In essence, the interplay of human perceptions, expectations, and interactions can shape the behavior of domesticated animals in a species-specific manner. As a result, the effects related to the behavioral ecology of the ancestors of the domesticated species and their domestication histories could be partially masked. 

Thus, the primary objective of our current study was to conduct a cognitive comparison between two domesticated species that have had limited human socialization. To this scope, we selected a group of goats and dogs that were similarly raised and kept, with opportunities for socialization within their respective species. However, in their daily routines, their interactions with humans were kept at a minimal level, primarily limited to a single man (caretaker) responsible for daily cleaning and feeding. This unique approach was particularly valuable as, unlike the majority of companion and working dogs typically used in similar studies, these dogs had experienced minimal human contact while being bred and housed in the facility. Consequently, their behavior during the problem-solving task could be effectively compared with that of goats, which were primarily kept for milking but still had moderate human interaction. Furthermore, the animals were tested in the presence of unfamiliar individuals to minimize the effects associated with a potential attachment bond formed with the caretaker. 

We employed the impossible task paradigm, which has previously been used in the studies comparing dogs and cats [11], and dogs and pigs [12]. 

Anticipated in our study is the likelihood that dogs, owing to their history of close cooperation with humans and behavioral ecology rooted in cooperative interactions among individuals, will demonstrate behaviors oriented towards seeking assistance and communication with humans in the impossible task paradigm. In contrast, goats, primarily domesticated for utilitarian purposes rather than companionship, may not possess the same level of inclination or experience in engaging with humans in this scenario. Moreover, goats, as prey species, can experience extra difficulty in interacting with humans, who carry typical predator features (although goats are typically used through human manipulation for the milking procedures).

## 2. Materials and Methods

### 2.1. Animals

In this study, we enrolled a total of 14 female dogs (*Canis lupus familiaris*), with a mean age of 5 years (± SD 1.75), and 14 female goats (*Capra aegagrus hircus*), with a mean age of 2.79 years (± SD 1.42). All the dogs were of the “Australian Cattle Dog” breed, while all the goats belonged to the “Camosciata delle Alpi” breed. All animals were reared in a farm in Cassino, Italy, called “Eugenia Palumbo’s Funky Farm”.

Both species were reared using a gentle approach. They lived in outdoor pens and only had contact with humans during routine caregiving activities and periodic veterinary checkups (such contact was nearly absent during the initial development phase, which was entirely entrusted to the mothers). The goats were also handled for milking purposes. Both the dogs and goats had the opportunity to leave their pens daily and freely interact with the environment in small groups. The interspecies interactions were occasional. The dogs were let out for approximately one hour each day and were typically fed in the afternoon. The goats, on the other hand, were let out of their pens for grazing activities in the morning for about four hours. They also received a food supplement consisting of a mix of barley seeds, triticale, and field beans, with 500 g divided between the morning and the evening. Interactions between the dogs and goats were limited.

### 2.2. Testing Procedure

**Figure 1:**
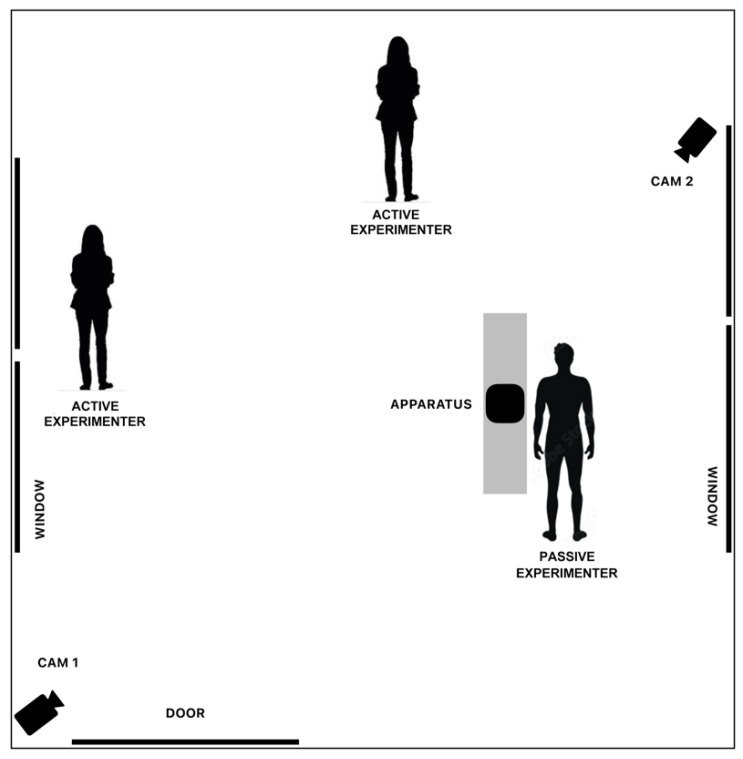
Experimental room equipped with two cameras (in the corners) and the apparatus (in the middle). A stranger was positioned on the one side of the apparatus.

Each subject participated in a single session. The experiments were conducted in the afternoon, with each animal individually tested in an unfamiliar room located near the fences. The room had an approximate area of 30 m^2^ and was equipped with two cameras (Sony^®^ HDR CX115, Sony^®^ HDR-PJ260VE, Tokyo, Japan) placed in different corners of the room (Figure 1). 

**Figure 2:**
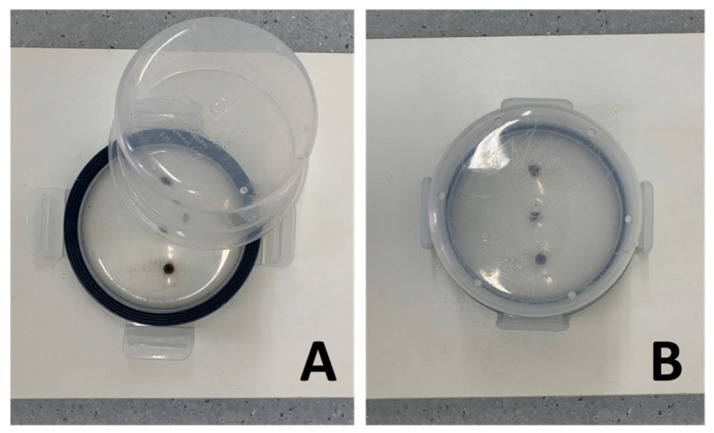
The image shows a detail of the food container used during the testing procedure as it appears in both the solvable phase (**A**) and the unsolvable phase (**B**).

The experimental apparatus (Figure 2), which the animals had never encountered before, was positioned towards one of the walls at approximately one-third of the length of the side wall.

The animals selected for testing were gently brought directly into the testing room by the caretaker. Inside the room, there were three experimenters. Two females actively managed the animals with a friendly approach to establish a positive association, while a male passive experimenter maintained a distance and did not handle the food during the familiarization process, nor interacted with the animals. All experimenters were unfamiliar with the animals. The active experimenters approached the animals using appealing food: würstel for the dogs and oat seeds for the goats, as recommended by the caretaker. Initially, the food was either offered by hand or placed on the floor away from the animals. As the familiarization progressed, the experimenters gradually moved the food closer to the experimental apparatus, ultimately giving it over the apparatus itself. Throughout this process, the experimenters had the opportunity to interact with the animals if they accepted their contact. The familiarization period lasted for 15 min, during which the animals became accustomed to the experimental apparatus and were subsequently subjected to the impossible task paradigm.

The experimental apparatus utilized in this study consisted of a plastic feed container positioned on a rectangular wooden platform. The lid of the feed container was securely attached to the platform, while the container itself was inverted and placed on the lid’s tracks. The wooden platform was fixed to the floor using double-sided adhesive tape to ensure stability. All components of the experimental apparatus were thoroughly washed with a mildly scented, non-toxic disinfectant after each test. 

The testing procedure consisted of three solvable trials, followed by an unsolvable trial. In the solvable trials, the animals had the opportunity to obtain the food by moving the container. However, in the unsolvable trial, the container was locked on the upside-down lid. Throughout the trials, the passive experimenter remained in close proximity to the apparatus, maintaining a constant gaze ahead and ignoring the animal’s presence and actions, even if the animal attempted to make contact. The two active experimenters managed the animals during the different phases. One experimenter lured the animal away from the apparatus, while the other placed the food below the plastic container in preparation for the subsequent trial. After completing the three solvable trials, the impossible phase commenced, lasting for one minute. During this phase, the two active experimenters moved towards separate walls, turned their backs to the animals, and ignored their presence. Any animals that refused food or failed to approach or manipulate the apparatus in all three solvable trials were excluded from the testing procedure.

The experimental study was conducted in compliance with ethical standards and was approved by the Ethical Committee for Animal Experimental Procedures (CESA) of the University of Naples Federico II (Protocol Number 2017/0025509). This study adhered to all relevant international, national, and institutional guidelines for the care and use of animals, following the principles outlined in the Declaration of Helsinki.

### 2.3. Behavioral Assessment

The recorded footage was analyzed using the Solomon Coder beta^®^ 16.06.26 software (ELTE TTK, Hungary). A researcher coded the animals’ behaviors of the unsolvable trials using a specific ethogram provided in Table 1. A continuous sampling method was employed to record the behaviors of interest throughout the trial. The behaviors that were recorded included the visual and tactile approaches to the apparatus, the experimenters, and the door. Additionally, visual and olfactory exploration, as well as stress-related behaviors, were recorded.

The data collected included information on behavior duration, frequency, and latency. To ensure the accuracy and reliability of the data, an inter-observer reliability assessment was conducted. This involved comparing the results obtained by a second independent coder on 20% of the samples. The inter-observer reliability agreement ranged from 97% to 99% depending on the specific variable being considered.

### 2.4. Statistical Approach

To address the issue of excessive zeros in the behavioral data due to animals not expressing the entire behavioral repertoire listed in Table 1, we grouped individual behaviors directed towards the same target into specific behavioral categories. This resulted in the creation of the following variables:

“*Apparatus*”: behaviors directed towards the apparatus, indicating a willingness to solve tasks.

“*Door*”: behaviors directed towards the door.

“*Exploration*”: behaviors related to visual and olfactory explorations.

“*Stress*”: behaviors that include stress-related signals.

“*Other*”: behaviors that could not be assigned to any of the previously mentioned categories.

“*A-experimenters*”: behaviors directed towards the active experimenters.

“*P-experimenter*”: behaviors directed towards the passive experimenter.

It is important to note that the experimenters were kept separate in this analysis, as their roles and interactions with the animals differed. The active experimenters interacted with the animals before the impossible phase, while the passive experimenter remained aloof; however, during the impossible phase, the active experimenters positioned themselves far from the animals, while the passive experimenter remained close to the apparatus where the animals spent most of their time. By separating these variables, we were able to assess the distinct effects of each experimenter’s presence on the animals’ behavior.

Due to the smaller sample size, a non-parametric statistical approach was adopted for the analysis. Additionally, to assess the distribution of the behavioral parameters, the Kolmogorov–Smirnov test was conducted, which revealed that most of the variables were not normally distributed.

As a first step, the Mann–Whitney U test was utilized to examine for potential statistical differences in the duration, frequency, and latency of the variables between the two species. 

Given the significant difference in age between the species (U = 34.000; Z = −2.866; *p* = 0.004), we conducted further analysis using generalized linear models (GzLMs) to examine whether the behaviors of dogs and goats, in terms of the duration, frequency, and latency of all behavioral variables, could be predicted by age. In these models, *Species* (i.e., dogs and goats) was set as the explanatory factor and *Age* as the covariate. We tested the main effects of *Species* and *Age*, as well as the first-level interaction between the *Species* and *Age*. A linear model was chosen for the GzLM analysis. Statistical analysis was performed using IBM SPSS Statistic version 26 (IBM Corp., Armonk, NY, USA).

## 3. Results

Out of the total number of animals, eight dogs and eight goats (57%) successfully met the requirements and proceeded to the impossible phase of the test. However, six dogs and six goats had to be excluded from the study. The animals that were not able to complete the test (43% of both dogs and goats) exhibited significant discomfort in the experimental room. This discomfort was manifested by their refusal of food (29% of dogs and 14% of goats), avoidance of social contact (14% of dogs and goats), or a combination of both (14% of dogs and 7% of goats). In some cases, although the subjects allowed themselves to be approached and touched, they did not demonstrate the willingness to approach the apparatus, which prevented them from performing the test (14% of dogs and 21% of goats).

**Figure 3:**
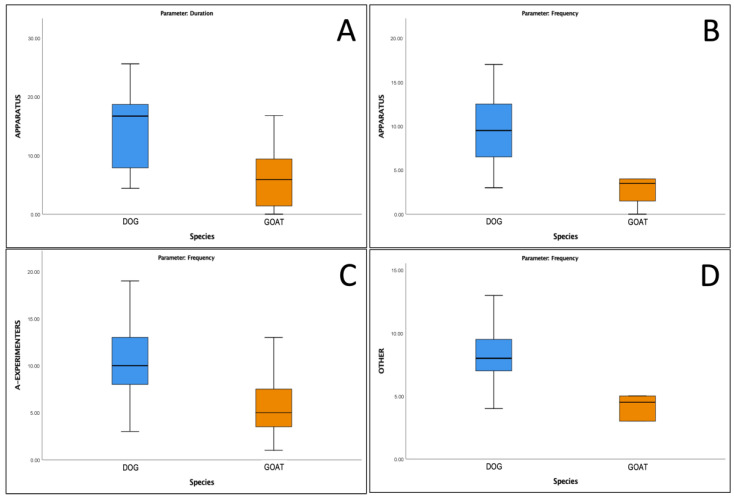
Graphics of significant species differences, as reported in Table 2. The box plots compare species according to the *Apparatus* duration (**A**) and frequency (**B**), *A-experimenters* frequency (**C**), and *Other* behaviors frequency (**D**).

According to the Mann–Whitney U test, there were significant differences between the two species in several behavioral parameters (Table 2). Dogs demonstrated a significantly longer duration (Figure 3A) and higher frequency (Figure 3B) of *Apparatus* compared to goats. Dogs also exhibited a higher frequency of *A-experimenters* (Figure 3C). Furthermore, dogs displayed a significantly higher frequency of *Other* behaviors compared to goats (Figure 3D).

The GzLMs investigated the impacts of *Species* and *Age* on the frequency, duration, and latency of behavioral variables (*A-experimenters*, *P-experimenter*, *Apparatus*, *Door*, *Exploration*, *Other*, and *Stress*). The results revealed that the full model significantly improved the fit over a null model in five instances.

*A-experimenters* frequency (omnibus test: χ2 = 14.643, *p* = 0.002): the GzLM revealed a main effect of *Species* (β = 12.255, χ2 = 11.672, *p* = 0.001), indicating that dogs were more likely to interact with the A-experimenters more frequently compared to goats.

*A-experimenters* latency (omnibus test: χ2 = 7.551, *p* = 0.056): the GzLM showed a negative main effect of *Age* (β = −0.909, χ2 = 4.764, *p* = 0.029), indicating that older animals (both dogs and goats) were more likely to rapidly interact with the *A-experimenters*.

*Apparatus* frequency (omnibus test: χ2 = 13.562, *p* = 0.004): the GzLM showed a main effect for *Species* (β = 10.200, χ2 = 7.455, *p* = 0.006), indicating that dogs were more likely to have more frequent interaction with the *Apparatus* compared to goats.

*Door* latency (omnibus test: χ2 = 11.608, *p* = 0.009): the GzLM reported a positive main effect for *Age* (β = 6.826, χ2 = 4.628, *p* = 0.031), suggesting that older animals (both dogs and goats) had a higher probability of interacting later with the *Door*. There was also a negative interaction between *Species* and *Age* (β = −12.035, χ2 = 9.163, *p* = 0.002), suggesting that older dogs had a high probability to rapidly interacting with the *Door*.

*Other* frequency (omnibus test: χ2 = 16.238, *p* = 0.001): the GzLM revealed a main effect of *Species* (β = 8.211, χ2 = 15.400, *p* = 0.000), indicating that dogs were more likely to perform *Other* behaviors more frequently compared to goats. There was also a negative interaction between *Species* and *Age* (β = −0.972, χ2 = 4.108, *p* = 0.043), suggesting that younger dogs had a lower probability of performing high-frequency *Other* behaviors.

The GzLMs with all the other variables and ethological parameters did not yield significant effects.

## 4. Discussion

The aim of this study was to investigate whether domesticated dogs and goats, raised under conditions with limited human socialization, exhibit different propensities for social interaction with unfamiliar individuals. Using the “impossible task” paradigm, we investigated the behavior of dogs and goats when confronted with an unsolvable task, hypothesizing that dogs, due to their long history of selective breeding for human interaction (and the behavioral ecology of their ancestors), would exhibit higher levels of social engagement with humans compared to goats. The results of our study indicated that dogs showed a significantly higher frequency of approaching the active experimenters compared to goats. Conversely, no significant differences among species were observed in their behavior towards the passive experimenter, who had no prior social interaction with the animals. These findings could support our hypothesis that dogs demonstrate a heightened propensity for social interaction with humans in comparison to goats; however, this tendency appeared to be specifically directed towards the active experimenters. However, considering the infrequent interactions of the subjects with the passive experimenter, it is possible that their human-directed engagement, primarily with the earlier active experimenters, may have been driven by factors other than seeking assistance with the apparatus, such as seeking comfort or an exit from the room. This suggests a need for further research to explore the complex motivations behind these interactions.

Regarding the observed species differences, they can be attributed to several factors. Firstly, dogs have been bred for thousands of years to be companions and working partners of humans, resulting in a close and cooperative relationship with friendly humans [2], even when they are unfamiliar. In contrast, the domestication history and selective breeding of goats have not placed as much emphasis on human interaction [25].

It can be considered that the ecological and social contexts of the ancestors of dogs and goats could have played a role in shaping their social cognitive abilities. Wolves, the ancestors of dogs, are highly social animals with complex social dynamics and cooperative behaviors [2,26]. This social structure likely played a crucial role in the development of dogs’ social cognitive skills, as they evolved to communicate and collaborate with other pack members, including humans [27]. In contrast, the ancestors of goats, wild goats, had a more independent and less cooperative-oriented lifestyle [25]. The domestication of goats may have introduced some changes in their social cognitive abilities, but their natural predispositions may still influence their level of interaction and cooperation with humans. A similar reasoning can also be applied for the study comparing horses and dogs [8], puppies and piglets [9,12], and dogs and cats [11], although these subjects were socialized with humans.

While this reasoning may seem logical and suitable for drawing conclusions, it solely relies on the frequency of human-directed behaviors. However, when considering our data regarding both the duration and latency of these behaviors, it becomes evident that this argument lacks complete support. Indeed, we found no significant interspecies differences in the duration and latency of human-directed behaviors. Rather, it appears that goats tend to exhibit higher durations and shorter latencies in human-directed behaviors compared to dogs, despite the lack of statistical significance (see Table 2). The higher occurrence of human-directed behaviors in dogs may not solely stem from their eagerness to engage with humans but could also stem from their heightened behavioral flexibility. In fact, we noticed a notably greater frequency of behaviors unrelated to humans or task resolution in dogs compared to goats. This points towards dogs having a greater inclination to shift between diverse behaviors, consequently potentially amplifying the instances of human-directed behaviors. Considering these findings, we must cautiously interpret the enhanced frequency of human-directed behaviors in dogs compared with goats and avoid drawing strong conclusions about their enhanced willingness to interact with humans. Alternatively, we might have to assume that there are no significant differences between dogs and goats in their willingness to interact with humans. If this holds true, the logical inference would suggest that despite the diverse behavioral ecology of their ancestors, the domestication process could have driven both species towards similar prosocial propensities towards humans.

In our study, dogs displayed a significantly higher frequency and longer duration of interactions with the apparatus, indicating a stronger interest and engagement with the task compared to goats. In another comparative study on domesticated species, however, dogs were found to be less persistent in the impossible task paradigm when compared to cats [11]. The authors attributed this difference to the behavioral ecology of the ancestors of dogs and cats, as well as the influence of the domestication process. Unlike dogs, cats originate from solitary ancestors and were selected for their ability to work autonomously. Due to their solitary hunting background, it is expected that cats would display an increased persistence on tasks compared to dogs. On the other hand, goats, being herbivores, generally face fewer challenges in finding food compared to dogs. As a result, they might lose interest in the task earlier once they realize its difficulty, potentially explaining their lower level of interaction with the apparatus compared to dogs. It is worth highlighting that pigs share a closer behavioral ecology with goats rather than with cats. This might initially suggest a lower willingness to solve the task. However, contrary to this expectation, piglets displayed an increased persistence on the apparatus when compared to dogs [12]. This could be attributed to their encounter with food sources that require manipulation, such as digging them out from the ground with their snouts. These findings suggest that the cognitive abilities and problem-solving behaviors of domesticated species can be more nuanced and diverse than previously assumed based solely on their behavioral ecology.

Unfortunately, in the current research and previous comparative studies, there is no way to disentangle the relative weight of the domestication history and the behavioral ecology of the ancestors of the animals in shaping socio-cognitive skills.

It is essential to acknowledge that the present study was limited to a specific population of dogs and goats raised under similar conditions. Consequently, caution must be exercised when generalizing the results to all dogs and goats. Regrettably, our study had to contend with constraints imposed by the selection of experimental subjects, resulting in a limited sample size that could have potentially limited the results. Therefore, to solidify and expand on our findings, additional research with larger sample sizes and diverse breeds of both dogs and goats would be beneficial.

## 5. Conclusions

In conclusion, this study highlighted that dogs exhibited an elevated frequency of interaction with humans. Nonetheless, this isolated data point could be attributed to the inherent behavioral flexibility of dogs, as there were no distinctions in the duration and latency of human-directed behaviors between the two species. This implies that when dogs and goats experience under-socialization and interact with unfamiliar humans, their inclination for social interaction with humans may not exhibit significant differences. This suggests that the process of domestication, primarily centered around tameness, potentially drives both species towards a comparable socio-cognitive relationship with humans. In contrast, dogs consistently demonstrated a pronounced eagerness to engage in problem-solving tasks, which could be linked to their predatory nature.

## Figures and Tables

**Table 1:** Ethogram adopted for the analysis of behaviors.

Behavior	Description	Target
Visual approach	From a stationary position, the animal turns or lifts its head towards the target without approaching it.	Apparatus Experimenters Door
Tactile approach	The animal makes physical contact with the target by rubbing, nosing, pushing, licking, pawing, biting, and nibbling.	
Visual exploration	The animal engages in exploratory behavior by turning or raising its head to visually survey the environment, both while stationary and in motion.	Environment
Olfactory exploration	The animal engages in environmental exploration by sniffing while both stationary and in motion.	
Stress	Yawning, licking, shaking off, scratching, and locomotion (moving around the test arena without a specific direction). Some of these behaviors may be specific depending on the species being examined.	
Other	Behaviors that do not fall into any of the other predefined categories (such as galloping and rolling) or when the animals were in a blind point out of sight.	

**Table 2:** Statistical parameters, median, and interquartile ranges (values expressed in seconds) according to species. Significant differences are indicated in bold.

		DOG_Median [q1; q3]	GOAT_Median [q1; q3]	*U*	*Z*	*p*
A-experimenters	Duration	11.70 [7.95; 18.35]	16.00 [13.10; 22.00]	20.5	−1.21	0.227
	**Frequency**	10.00 [8.00; 13.50]	5.00 [3.25; 7.75]	12.5	−2.057	**0.04**
	Latency	4.90 [1.50; 6.30]	1.90 [1.65; 5.00]	24.5	−0.791	0.429
P-experimenter	Duration	3.80 [2.55; 4.35]	2.0 [0.80; 7.15]	21.5	−1.105	0.269
	Frequency	3.00 [2.00; 5.00]	1.50 [1.00; 4.75]	23	−0.966	0.334
	Latency	20.80 [10.30; 30.30]	12.90 [2.70; 40.50]	24	−0.84	0.401
Apparatus	**Duration**	16.70 [6.45; 16.70]	5.90 [0.80; 9.60]	10	−2.31	**0.021**
	**Frequency**	9.50 [6.25; 12.75]	3.50 [1.25; 4.00]	4.5	−2.912	**0.004**
	Latency	0.00 [0.00; 1.65]	0.00 [0.00; 0.00]	29	−0.462	0.644
Door	Duration	2.40 [0.30; 6.60]	6.80 [0.00; 12.30]	26	−0.64	0.522
	Frequency	2.50 [1.25; 3.75]	1.00 [0.00; 2.50]	18	−1.498	0.134
	Latency	20.60 [9.30; 34.60]	48.50 [20.70; 60.00]	16.5	−1.64	0.101
Exploration	Duration	3.20 [1.20; 5.80]	2.40 [0.20; 11.80]	29.5	−0.264	0.792
	Frequency	2.50 [1.00; 3.75]	1.00 [0.25; 3.75]	22.5	−1.032	0.302
	Latency	34.50 [13.65; 45.00]	35.00 [10.25; 57.50]	30	0.21	0.833
Other	Duration	9.10 [6.15; 15.10]	6.70 [3.40; 15.30]	27	−0.525	0.600
	**Frequency**	8 [6.50; 10.25]	4.5 [3.00; 5.00]	4.5	−2.943	**0.003**
	Latency	4.70 [1.55; 8.25]	2.50 [1.30; 7.70]	29.5	−0.263	0.793
Stress	Duration	0.00 [0.00; 5.30]	3.40 [0.55; 5.05]	22	−1.096	0.273
	Frequency	0.00 [0.00; 1.00]	1.50 [0.25; 3.75]	17.5	−1.604	0.109
	Latency	60.00 [14.25; 60.00]	26.10 [16.05; 58.70]	24	−0.877	0.380

## Data Availability

The datasets generated during and/or analyzed during the current study are available from the corresponding author upon reasonable request.

## References

[1] Zeder M.A. (2012). The Domestication of Animals. J. Anthropol. Res..

[2] Hare B., Brown M., Williamson C., Tomasello M. (2002). The domestication of social cognition in dogs. Science.

[3] Kaminski J., Nitzschner M. (2013). Do dogs get the point? A review of dog-human communication ability. Learn. Motiv..

[4] Agnvall B., Bélteky J., Katajamaa R., Jensen P. (2018). Is evolution of domestication driven by tameness? A selective review with focus on chickens. Appl. Anim. Behav. Sci..

[5] Beckman A.K., Richey B.M.S., Rosenthal G.G. (2022). Behavioral responses of wild animals to anthropogenic change: Insights from domestication. Behav. Ecol. Sociobiol..

[6] Udell M.A.R., Dorey N.R., Wynne C.D.L. (2010). What did domestication do to dogs? A new account of dogs’ sensitivity to human actions. Biol. Rev..

[7] Barrera G., Mustaca A., Bentosela M. (2011). Communication between domestic dogs and humans: Effects of shelter housing upon the gaze to the human. Anim. Cogn..

[8] McKinley J., Sambrook T.D. (2000). Use of human-given cues by domestic dogs (*Canis familiaris*) and horses (*Equus caballus*). Anim. Cogn..

[9] Gerencsér L., Pérez Fraga P., Lovas M., Újváry D., Andics A. (2019). Comparing interspecific socio-communicative skills of socialized juvenile dogs and miniature pigs. Anim. Cogn..

[10] D’Aniello B., Scandurra A. (2017). Impossible Task Paradigm. Encycl. Anim. Cogn. Behav..

[11] Miklósi Á., Pongrácz P., Lakatos G., Topál J., Csányi V. (2005). A comparative study of the use of visual communicative signals in interactions between dogs (*Canis familiaris*) and humans and cats (*Felis catus*) and humans. J. Comp. Psychol..

[12] Pérez Fraga P., Gerencsér L., Lovas M., Újváry D., Andics A. (2021). Who turns to the human? Companion pigs’ and dogs’ behaviour in the unsolvable task paradigm. Anim. Cogn..

[13] Topál J., Miklósi Á., Csányi V. (1997). Dog-human relationship affects problem solving behavior in the dog. Anthrozoos.

[14] Dobos P., Pongrácz P. (2023). Would You Detour with Me? Association between Functional Breed Selection and Social Learning in Dogs Sheds Light on Elements of Dog–Human Cooperation. Animals.

[15] Marshall-Pescini S., Passalacqua C., Barnard S., Valsecchi P., Prato-Previde E. (2009). Agility and search and rescue training differently affects pet dogs’ behaviour in socio-cognitive tasks. Behav. Processes.

[16] D’Aniello B., Scandurra A., Prato-Previde E., Valsecchi P. (2015). Gazing toward humans: A study on water rescue dogs using the impossible task paradigm. Behav. Processes.

[17] Scandurra A., Prato-Previde E., Valsecchi P., Aria M., D’Aniello B. (2015). Guide dogs as a model for investigating the effect of life experience and training on gazing behaviour. Anim. Cogn..

[18] Lazarowski L., Dorman D.C. (2015). A comparison of pet and purpose-bred research dog (*Canis familiaris*) performance on human-guided object-choice tasks. Behav. Processes.

[19] D’Aniello B., Alterisio A., Scandurra A., Petremolo E., Iommelli M.R., Aria M. (2017). What’s the point? Golden and Labrador retrievers living in kennels do not understand human pointing gestures. Anim. Cogn..

[20] Nawroth C., Brett J.M., McElligott A.G. (2016). Goats display audience-dependent human-directed gazing behaviour in a problem-solving task. Biol. Lett..

[21] Mastellone V., Scandurra A., D’Aniello B., Nawroth C., Saggese F., Silvestre P., Lombardi P. (2020). Long-Term Socialization with Humans Affects Human-Directed Behavior in Goats. Animals.

[22] Howell T.J., Bowen J., Fatjó J., Calvo P., Holloway A., Bennett P.C. (2017). Development of the cat-owner relationship scale (CORS). Behav. Processes.

[23] Riggio G., Piotti P., Diverio S., Borrelli C., Di Iacovo F., Gazzano A., Howell T.J., Pirrone F., Mariti C. (2021). The dog–owner relationship: Refinement and validation of the italian c/dors for dog owners and correlation with the laps. Animals.

[24] Borrelli C., Riggio G., Howell T.J., Piotti P., Diverio S., Albertini M., Mongillo P., Marinelli L., Baragli P., Di Iacovo F.P. (2023). The Cat–Owner Relationship: Validation of the Italian C/DORS for Cat Owners and Correlation with the LAPS. Animals.

[25] Nawroth C. (2017). Invited review: Socio-cognitive capacities of goats and their impact on human–animal interactions. Small Rumin. Res..

[26] Boitani L., Ciucci P. (1995). Comparative social ecology of feral dogs and wolves. Ethol. Ecol. Evol..

[27] Range F., Marshall-Pescini S., Kratz C., Virányi Z. (2019). Wolves lead and dogs follow, but they both cooperate with humans. Sci. Rep..

